# A. V. M. A.

**Published:** 1901-11

**Authors:** 


### A. V. M. A.

THE Executive Committee of the A. V. M. A. for the ensuing year as appointed by President Winchester covers pretty thoroughly various sections of the country and adds some new names to the body. Drs. D. E. Salmon, Chairman; H. D. Hanson, M. H. Reynolds, C. A. Carey, G. A. Johnson, T. E. Budd, and W. Horace Hoskins make up the committee. The following places are mentioned for the next meeting of the A. V. M. A.: Helena, Montana, strongly advocated by Dr. M. E. Knowles; Minnesota, by the veterinarians from that State; Columbus, Ohio, as well as Cleveland, have also been mentioned as suitable places for the meeting.

---

THE JOURNAL readers will excuse the late issue of the November number. The illness of the senior editor, Dr. Huidekoper, occurring in October, continued during the month of November, thus interrupting the return of the JOURNAL to its regular day of issue. His promised convalescence, we trust, will enable its putting forth the December number on time. We are very grateful for the many warm expressions of sympathy and good wishes for the senior editor, which we take this way of acknowledging, so that all may know of our receipt of the same.

---

IT is proper to state that no officer or employé of the Federal Government had any part in the preparation, publication, or distribution of the editorial that appeared in this JOURNAL on the attitude of the Central Short-horn Breeders' Association toward the tuberculin test.

